# Exploring the attitudes of mental health professionals toward recovery and peer support in Taiwan

**DOI:** 10.1186/s12888-025-06705-7

**Published:** 2025-03-22

**Authors:** Yen-Ching Chang, Meng-Wen Huang, Tzu-Hsuan Chuang, Su-Ting Hsu

**Affiliations:** 1 Department of Occupational Therapy, College of Medicine, National Cheng Kung University, Tainan, Taiwan; 2 Department of Rehabilitation Science, Jenteh Junior College of Medicine, Nursing and Management, Miaoli, Taiwan; 3 Department of Visual Arts, University of Taipei, Taipei, Taiwan; 4 Kaohsiung Municipal Kai-Syuan Psychiatric Hospital, Kaohsiung, Taiwan

**Keywords:** Recovery, Peer support services, Peer support workers, Mental illness, Professionals

## Abstract

**Background:**

While many Western countries have implemented mental health recovery-oriented and peer support services, they are still in the early stages of development in Taiwan, and the existing literature on professionals’ perspectives regarding recovery-related issues in non-Western contexts remains limited. This study investigates the perspectives of professionals from well-established psychiatric rehabilitation organizations in Taiwan on the concept of recovery and peer support.

**Methods:**

Data were collected through semi-structured interviews involving 19 professionals from nine organizations, which were then recorded and transcribed verbatim. Subsequently, the data were coded using Atlas.ti 8.0, followed by thematic analysis.

**Results:**

The analyses identified three main themes. First, attitudes toward recovery encompassed six subthemes: (1) coexisting with illness while finding personal fulfillment, (2) exercising one’s rights and taking personal responsibility, (3) maintaining hope throughout the recovery journey, (4) recognizing the non-linear nature of recovery, (5) acknowledging the individuality of each recovery path, and (6) affirming that recovery is achievable for all. Additionally, four types of peer support were identified: supportive peer, staff assistant, life assistant, and mental health workforce. Furthermore, perspectives on peer support workers included four subthemes: (1) attitudes toward people with mental illness, (2) the need for a robust career support system, (3) organizational and professional support, and (4) self-awareness of people with mental illness.

**Conclusions:**

This study is one of the few that explore the perspectives of professionals on recovery and peer support in non-Western contexts. Professionals from well-established psychiatric rehabilitation organizations in Taiwan embraced most mental health recovery principles, but inconsistencies were found regarding the applicability of recovery to different individuals. Peer support in Taiwan remains largely informal, with concerns primarily focused on environmental factors. However, one personal factor is also critical. People with mental illness in non-Western contexts may require more extensive preparation to serve as peer support workers.

## Background

Mental health recovery, as advocated by people with mental illness, involves actively participating in life decisions, overcoming the effects of mental illness, regaining control of one’s life, reintegrating into society, connecting with others, and developing personal goals and meaning within and beyond the limits of mental illness [1–3]. Recovery-oriented services are a person-centered care model designed to support people with mental illness in their recovery journey. These services focus not only on symptom management but also on housing, employment, education, and citizenship. The relationship between service providers and people with mental illness has shifted from a traditional and paternalistic model to an open and cooperative one, where professionals are “on tap, not on top.” Professionals are now partners, rather than sole experts, in treatment programs [4, 5].

In addition, advocates who had poor experiences receiving traditional treatments sought to develop self-help alternatives to replace conventional professional services [6]. Through their efforts, various peer support programs emerged, offering services such as peer support groups, crisis intervention, and drop-in centers [7]. Individuals with lived experiences of mental illness now have the opportunity to provide services to their peers and are known as peer support workers, peer support specialists, and consumer providers [8]. This paper uses the term “peer support workers” to refer to individuals in recovery who have undergone training and certification to perform tasks ranging from counseling and support services to clerical duties [8–10]. They share their life experiences to help others with mental illness gain a deeper understanding of their situations, reduce social isolation, obtain support and empathy, and increase hope. This role also allows people with mental illness to move beyond a passive patient role and become role models for others [11].

Research indicates that peer support services benefit both peer support workers and individuals receiving services. Peer support workers offering these services can overcome stigma, build confidence and hope, address fears of isolation, and enhance their working skills [12, 13]. Meanwhile, people with mental illness receiving peer support experience improvements in family and psychosocial functioning, reduced inpatient days, enhanced social skills, and increased hope [13–17]. Peer support services significantly contribute to the recovery journey of people with mental illness.

Globally, recovery-oriented services, which originated from Western contexts, have become the predominant approach in mental health care [18–20]. Similarly, peer support services are gaining momentum and are increasingly integrated into recovery-oriented care models [8, 21, 22]. While recovery-related literature in non-Western contexts is increasing [20], research on peer support services remains predominantly derived from Western countries [23, 24]. To further explore this emerging field, additional studies are needed in non-Western contexts [23, 25]. Moreover, professionals’ attitudes toward recovery are closely linked to the practical implementation of recovery-oriented approaches and the recovery process of people with mental illness [26, 27]. However, limited research has explored the perspectives of mental health professionals in non-Western contexts regarding recovery-related issues [28, 29]. In Taiwan, recovery-oriented services and related policies remain in their early stages, and formal peer support worker positions are largely nonexistent. Professionals well-versed in the concept of recovery and Taiwan’s mental health services can provide localized insights into this issue. Accordingly, this study investigates the perceptions of professionals from well-established psychiatric rehabilitation organizations in Taiwan on the concept of recovery and peer support.

## Methods

### Research context

Taiwan has 276 community psychiatric rehabilitation organizations supported by National Health Insurance, which include 98 day programs and 178 housing programs [30]. Currently, there is no certification mechanism specifically for recovery-oriented organizations. However, the concept of recovery has been integrated into the accreditation standards of psychiatric rehabilitation organizations, encouraging a gradual transition toward a recovery-oriented approach. Moreover, given that the concept of recovery originated in Western contexts and remains relatively novel to Taiwanese professionals, supervisors of well-established organizations in Taiwan typically play a pivotal role in guiding their staff and organizations toward embracing a recovery-oriented approach.

### Participants

The inclusion criteria for participants are as follows: professionals who work in psychiatric rehabilitation organizations, possess knowledge of recovery, and have experience implementing recovery-oriented services. The study employed snowball sampling to recruit participants. Researchers first interviewed several experienced professionals who were known to have been engaged in recovery-oriented services, and requested them to recommend other potential candidates. In addition, three members of accreditation committees, who have conducted extensive visits to evaluate various psychiatric rehabilitation organizations in Taiwan, were consulted to recommend benchmark organizations. These organizations were strategically chosen from different regions across Taiwan to ensure diverse representation. Subsequently, researchers reached out to the supervisors of selected organizations, requesting recommendations for experienced staff to participate in the study.

### Data collection

This study received approval from the institutional review board of the first author’s university. Researchers contacted professionals or organizational supervisors to schedule interviews and provided the interview guide in advance. Trained interviewers (Y.C.C. and M.W.H.) met with participants in meeting rooms at their workplaces to introduce the study. After providing informed consent, participants took part in individual and semi-structured interviews. Each interview lasted 60 to 90 min and was audio-recorded and transcribed verbatim. Portions of the interview data have been previously published [29]; this study presented professionals’ perspectives on recovery and peer support. The interview guide was developed based on recovery literature [31, 32] and the research objectives. Participants were questioned about their attitudes toward recovery, the types of peer support they knew in Taiwan, and their perceptions of peer support workers. Data collection concluded upon reaching data saturation, signifying that no new information was being obtained. During the collection period, no participants dropped out of the study.

### Reflexivity

Both interviewers are female occupational therapists: one holds a PhD, and the other is a graduate student. Their professional background enhances their understanding of mental health recovery. Nevertheless, the interviewers ensured that their perspectives did not overshadow the participants’ narratives throughout the study. Furthermore, no supervisor-subordinate relationship existed between the interviewers and participants. The interviewers prioritized establishing equal relationships with participants to encourage them to freely express their genuine thoughts.

### Data analysis

This study utilized thematic analysis with an inductive approach [33, 34], and data were coded using Atlas.ti 8.0 (Scientific Software Development, Berlin, Germany). Two researchers reviewed the transcripts line by line to generate codes. Subsequently, they discussed, combined, and organized the codes to outline preliminary themes. The research team reviewed these codes and themes until a consensus was reached. Finally, clear definitions for each theme were then generated to confirm the structure. To mitigate bias stemming from the researchers’ personal biases and values, verbatim transcripts and summaries were sent to each interviewee for confirmation, ensuring consistency between interviewees’ expressions and researchers’ interpretations. The participants reported no disagreements.

## Results

This study interviewed 19 professionals from nine psychiatric rehabilitation organizations in Taiwan, including seven day programs and two housing programs. Only one organization had experience in implementing a peer support training program. Participants were aged 23–62 years and had between 1.5 and 28 years of experience in mental health services across various disciplines (Table 1). The analyses identified three main themes: (1) attitudes toward recovery, (2) types of peer support in Taiwan, and (3) perspectives on peer support workers (Table 2).

**Table 1:** Participant characteristics

	*N* = 19
Gender, n (%)	
Female	15((79%)
Education, n (%)	
Bachelor’s degree	7 (37%)
Master’s degree or above	12(63%)
Profession, n (%)	
Psychiatrist	2(11%)
Nurse	5(26%)
Occupational therapist	5(26%)
Social worker	5(26%)
Case manager	2(11%)
Location	
Northern Taiwan	4(21%)
Central Taiwan	3(16%)
Southern Taiwan	8(42%)
Eastern Taiwan	4(21%)
Age (years), mean ± SD	42.42 ± 8.96
Working experience in mental health services (years), mean ± SD	15.55 ± 8.08

**Table 2:** Overview of themes and subthemes

Themes/Subthemes	Definitions of subthemes
1. Attitudes toward recovery	
Coexisting with illness while finding personal fulfillment	People with mental illness can live with symptoms and have a satisfying life
Exercising one’s rights and taking personal responsibility	People with mental illness have the right to make decisions and take responsibility
Maintaining hope throughout the recovery journey	Hope is an important component of recovery for people with mental illness
Recognizing the non-linear nature of recovery	The recovery process is non-linear, with fluctuations. People with mental illness may experience setbacks but keep moving forward
Acknowledging the individuality of each recovery path	Each individual with mental illness has a unique recovery process
Affirming that recovery is achievable for all	Every individual with mental illness can recover by pursuing individualized recovery goals
2. Types of peer support in Taiwan	
Supportive peer	People with mental illness have friendships with peers. They help each other naturally
Staff assistant	People with mental illness can assist the staff. Generally, they do tasks designated by staff, without pay
Life assistant	People with mental illness help peers’ daily living activities. They may receive rewards for their assistance
Mental health workforce	People with mental illness who are qualified for positions within psychiatric rehabilitation organizations or associations can become full-time or part-time employees
3. Perspectives on peer support workers	
Attitudes toward people with mental illness	Negative attitudes toward people with mental illness impede the development of peer support services
The need for a robust career support system	A comprehensive system is necessary for implementing peer support services
Organizational and professional support	Support from organizations and professionals is critical to the success of peer support workers
Self-awareness of people with mental illness	People with mental illness need to know themselves well to be qualified for peer support workers

### Theme 1: attitudes toward recovery

Overall, participants demonstrated a positive understanding of recovery, which encompassed six themes: (1) coexisting with illness while finding personal fulfillment, (2) exercising one’s rights and taking personal responsibility, (3) maintaining hope throughout the recovery journey, (4) recognizing the non-linear nature of recovery, (5) acknowledging the individuality of each recovery path, and (6) affirming that recovery is achievable for all.

#### Coexisting with illness while finding personal fulfillment

Participants emphasized that recovery was not defined solely by symptom severity or functionality. People with mental illness could lead fulfilling lives even if their symptoms persisted.

*“Their symptoms will stay with them throughout their lives, but we can help them to make their lives more satisfying and reach a more comfortable state. When they reach this state, they are on the way to recovery*.” (Nurse K)

*“They continue to strive in their lives, and I feel that they have already demonstrated this sense of meaning (recovery). In fact, this meaning is something they interpret for themselves.”* (Social Worker I)

#### Exercising one’s rights and taking personal responsibility

Participants emphasized that recovery was a human right. People with mental illness have the right to determine the life they want to live, and they know what they need best. Recovery allows people with mental illness to truly control their rights, make choices for themselves, and have the courage to take on the consequences of their decisions.

*“Once a choice is made, we must take responsibility for both the good and the bad. It is not about placing blame on others when things go wrong. They (people with mental illness), too, need to learn to take responsibility, just as we do, in order to grow.”* (Case Manager J)

*“Let them be self-aware, then understand themselves, and finally make their own decisions. They should be brave enough to make their own decisions, to speak up, to implement these choices, and finally take responsibility.”* (Psychiatrist A)

#### Maintaining hope throughout the recovery journey

Hope was viewed as a central component of recovery. Participants believed that hope and positive beliefs significantly impacted recovery, even if progress was incremental. People with mental illness could continue progressing with a sense of hope.

*“Although he has mental illness, he has hope. I think the spirit is quite important and will affect a person’s actions and thoughts.”* (Nurse S)

*“Recovery for individuals can involve functional performance in daily life. It might be something as small as a single event in their life—like progressing from not being able to do something to mastering it—or even just making a small improvement. All of these count as recovery.”* (Occupational Therapist D)

#### Recognizing the non-linear nature of recovery

Drawing from their experiences, participants recognized that the recovery process is not linear, and potential relapses or periods of functional decline should be acknowledged and accepted.

*“Last year, we referred a job to a client. We’ve provided counselling to him several times. He has fallen ill at work, been hospitalized, and then returned. So, at times, we say… he tried, he may fall or get sick, but he will still recover!”* (Nurse P)

*“In the recovery journey, he goes through the process of falling ill and rising again to gradually understand, “Ah, so this is who I am. Yes, this is the process.”* (Occupational Therapist E)

#### Acknowledging the individuality of each recovery path

Participants understood that recovery looks different for each individual, and each individual has differing characteristics, life expectations, and goals. Their recovery process and goals are therefore individualized, requiring professionals to respect and tailor their support accordingly.

*“Recovery does not necessarily mean going to work. You can do volunteer work, maintain a regular daily routine, or help take care of family members. The so-called recovery is, in my opinion, different from one person to another.”* (Occupational Therapist N)

*“Recovery must be tailored to each individual, considering their unique characteristics. For those with higher functioning, it might mean being able to work independently and maintain autonomy. For others, recovery might simply mean being able to avoid acting on auditory hallucinations. I think even achieving such a state can be considered a significant step toward recovery for them.”* (Nurse G)

#### Affirming that recovery is achievable for all

Some participants noted challenges in applying recovery principles to people with mental illness who had difficulty in accepting their condition, had unstable symptoms or medication compliance, or had poor cognitive function.

*“They must have expectations for themselves and possess basic insight into their condition, along with a willingness to manage their issues consistently. Only then can they begin to engage with the concept of recovery.”* (Case Manager J)

However, other participants believed that the principles of recovery applied to everyone. Everyone with mental illness has the opportunity to recover, and there may be different goals at different stages of the illness.

*“I think recovery can be used for everyone, but the operated elements will be different. For example, when a client is in a situation that is easier to discuss with, we can talk about empowerment, or if he is more autonomous, I will let him be more independent...and if he is in an acute phase...I will pass on a hope to him.”* (Social Worker O)

### Theme 2: types of peer support in Taiwan

Qualitative data indicated that peer support in Taiwan can be divided into four groups: supportive peer, staff assistant, life assistant and mental health workforce.

#### Supportive peer

Several participants stated that peers developed friendships naturally, which led to the formation of social networks and mutually supportive behaviours.

*“I have heard that they went out to sing karaoke or had dinner together. Even at the beginning of this year, two members went on a self-guided trip to Japan. (Nurse S)*

*When your roommate is sick, will you accompany him? We didn’t ask him to do so! But he was right there. The sick person was his roommate, his peer and his colleague.”* (Social worker B)

#### Staff assistant

People with mental illness can also take on roles as staff assistants, engaging in tasks such as writing meeting records, assisting peers in activities, and collaborating with staff to develop course content. These positions are typically voluntary.

*“We let members with better function take others with poor cognitive function to do easy activities, such as brain exercises and basic work training. He takes the role of team leader.”* (Social worker L)

#### Life assistant

Some people with mental illness are willing to assist their peers in daily living activities, such as showering, medication management, and accompanying them to medical appointments. They may receive payment from peers’ families or organizations, as noted by one participant:

*“He can help peers who have lower functioning. We told the families of those he helped that, because he helped their relatives, they might want to subsidise his transportation or provide a little reward. Although the amount of money was not high, the client was happy to have the opportunity to make money and help others, and he increased his sense of achievement.”* (Nurse G)

#### Mental health workforce

People with mental illness can secure positions within psychiatric rehabilitation organizations or mental health-related associations, either delivering services or managing administrative tasks. These roles were not initially designed for people with mental illness. However, their qualifications and familiarity with the organizations/associations led to their employment within the unit.

*“One client had a nursing background and got sick. After she recovered, she was hired by the organization to work as a nurse. Since she was a person in recovery, she knew what other peers went through during the recovery process and what they really cared about in their hearts. So, she can talk to clients about these things better than other staff.”* (Social worker L)

### Theme 3: perspectives on peer support workers

Some participants, particularly non-supervisors, were unfamiliar with the concept of peer support workers, necessitating the researcher to introduce this new job position. Participants highlighted the benefits of peer support workers, such as mutual understanding among peers and the sense of hope they bring. However, they also identified four considerations for promoting peer support services: attitudes toward people with mental illness, the need for a robust career support system, organizational and professional support, and self-awareness of people with mental illness.

#### Attitudes toward people with mental illness

The participants noted that both society and organizations harboured low trust in people with mental illness, potentially affecting their self-efficacy and employment prospects.

*“The attitude of our entire society toward people with mental illness is that people with mental illness cannot be peer support workers... Can the public trust them? Even people with mental illness don’t believe they can make it because that’s how society treats them.”* (Nurse K)

*“I think Taiwan is medically oriented. There is a lack of concept of equal rights in medically oriented services and professionals think people with mental illness are patients. Our hospital still feels that you (professionals) can’t leave him (a client) alone (to do tasks) because they can’t take the responsibility.”* (Occupational therapist E)

Additionally, the mindsets of professionals play a crucial role. They need to acknowledge the evolving role of people with mental illness from patient to colleague and be open to collaborating with peer support workers:

*“If you (professionals) can’t appreciate and understand these things (peer support), then you can’t let every person with mental illness be your colleague, right? Real peer support is impossible to achieve.”* (Psychiatrist A)

#### The need for a robust career support system

The participants acknowledged the value of peer support workers but highlighted the lack of a robust system to implement peer support services in Taiwan. Currently, there are unclear job duties and role positioning of peer support workers, insufficient training and practice courses, and the absence of licence regulation. They emphasized that establishing a solid system for peer support services would help the public recognise the contributions of peer support workers.

*“If they want to become peer support workers, do they need to start as work assistants, progress to supportive job positions, and then gradually transition to working independently? Is there a sound system for this?”* (Nurse G)

*“How do we define them (peer support workers)? Does accompanying someone to a doctor’s appointment count as a professional service?”* (Social worker B)

#### Organizational and professional support

Some participants expressed their inability to initiate new services due to a lack of manpower and resources, despite believing in the potential benefits for people with mental illness.

*“The organization needs to make the effort to train the client… it may be suitable for organizations which have sufficient ability and time to do this.”* (Social Worker L)

The participants also emphasized the critical need for professional support. They suggested that peer support workers may require assistance and support from professionals and their tasks should be carefully selected to avoid overwhelming them.

*“Ordinary people may have the ability to withstand work requirements and reactions from clients with mental illness. If you want people with mental illness to do such work, I don’t think it’s impossible; however, they should be given more support.”* (Case Manager J)

#### Self-awareness of people with mental illness

The participants emphasized the importance of self-awareness, self-belief, and a sense of responsibility for people with mental illness to serve effectively as peer support workers.

*“We have been dedicated to one thing recently, which is to make them self-aware, understand themselves, and finally make their own decisions. Let them be brave enough to decide to speak up, implement the plan, and finally take responsibility.... If they cannot do these things, they cannot do peer support.”* (Psychiatrist A)

*“I hope to try this (peer support worker), but I feel it is quite challenging. For them, the key issue is whether they possess a sense of responsibility. I believe there is still a need to cultivate this sense of responsibility to ensure that they have it and can act accordingly.”* (Occupational Therapist N)

## Discussion

In this study, 19 professionals from well-established psychiatric rehabilitation organizations in Taiwan were interviewed to explore their perceptions of recovery and peer support. Overall, participants held positive opinions about recovery, recognizing that people with mental illness can reintegrate into society, pursue their goals, make decisions, and take responsibility for their own lives, even while managing symptoms. Recovery was acknowledged as a personalized, lengthy, and non-linear process, requiring sustained hope throughout the journey. While previous studies reported uncertainty among professionals regarding the concept of recovery in Western contexts [10, 35, 36], this study observed positive attitudes that may be attributed to the participants’ familiarity with the concept of recovery and their practical experience in implementing recovery-oriented services. Similarly, Kuek et al. noted that Singaporean professionals emphasized symptoms and medication in their perspectives on recovery [28]. In contrast, our findings revealed that professionals from Taiwan’s well-established psychiatric rehabilitation organizations embraced most mental health recovery principles. Their understanding, shaped by Western literature, aligns closely with existing recovery models [1, 2, 37].

However, inconsistencies were found regarding the applicability of recovery. While most professionals believed that recovery could benefit everyone, some expressed reservations, suggesting that certain conditions must be met. This aligns with previous concerns that individuals with poor insight, low medication adherence, or in acute phases of illness may not be suitable for recovery-oriented approaches [5, 32, 38]. This finding highlights a challenge for professionals: how to tailor services for individuals with varying needs, which requires particular attention in non-Western settings. One potential solution is for the government and organizations to involve experienced professionals in providing practice-based training courses. Through case discussions and on-site observations, professionals can develop a deeper understanding of recovery-oriented services [29]. In addition, training that involves personal stories from people with mental illness can help expand professionals’ perspectives and understanding of this population [39].

Peer support services are well-established in Western countries and contribute positively to mental health services. Fortuna and Solomon (2022) delineated four forms of peer support in Western countries: (1) peer-delivered self-help, (2) peer-run services, (3) peer partnerships and (4) peers in recovery as employees [40]. Except for peer-delivered self-help, which is generally voluntary, other forms of peer support were provided by peer support workers from different types of organizations. The current study also identified four types of peer support: supportive peer, staff assistant, life assistant, and mental health workforce. However, in Taiwan, most peer support occurs informally and voluntarily. Formal job positions for peers are nonexistent, and only a few receive formal training.

In Chinese culture, Hong Kong stands out as a pioneer in peer support services and actively implements training programs for peer support workers. These programs offer training courses that enable them to provide services such as warmlines and vocational support. Research has also demonstrated the growth of peer support workers and positive changes in service users following the receipt of services [41–43]. By comparison, Taiwan has begun training peer support workers. Some units initiate peer training with non-official funding, providing services including warmlines, case management, and vocational training. Despite being in a nascent stage, research affirms the benefits of peer support services for both peers and service recipients in Taiwan [44, 45]. Additionally, although no formal peer support worker positions exist, the Ministry of Health and Welfare in Taiwan has made efforts to increase peer support resources. Currently, there is one accredited clubhouse, the Eden Clubhouse, in Taiwan, and plans have been announced to establish 49 ‘quasi-clubhouses’ from 2021 to 2025 [46], providing alternative services for people with mental illness in the community. This represents a significant achievement.

The results revealed four subthemes regarding the perspectives on peer support workers. The first three subthemes related to the environmental factors – attitudes toward people with mental illness, the need for a robust career support system, and organizational and professional support – and the fourth subtheme related to personal factors – self-awareness of people with mental illness. Our participants – drawn from recovery-oriented organizations – showed positive attitudes toward the new position of peer support workers and recognised their strengths. However, they also noted that negative attitudes toward people with mental illness, from both society and within organizations, and from professionals and peers, could hinder the development of peer support services. The existing literature indicates that some professionals perceive peer support workers as relatively vulnerable individuals requiring protection and express concern about their professional capabilities [13, 41, 47]. Peer support workers have also voiced concerns about being negatively labelled by colleagues and sometimes even being seen as patients in the workplace [48–50]. Encouragingly, research also indicates that as professionals and peer support workers collaborate over time, they tend to develop more positive attitudes toward peer support services and recognise their value [42, 51].

Participants reported that Taiwan currently lacks a robust system outlining training, certification mechanisms, and the scope of responsibilities for peer support services. Moreover, there is no policy incentive for organizations to establish similar positions or offer relevant support. Although participants had limited practical experiences in peer support services, they outlined potential concerns consistent with previous studies [24, 50, 52], including role definition, training, resource, and professional support. In terms of role definition, some studies have identified challenges in transitioning between the roles of peers as service providers and recipients, defining the role of peer support workers for professionals, and distinguishing boundaries between peer support workers and professionals as well as between peer support workers and service users [24, 50, 53]. Addressing these issues requires the Taiwanese government to delineate clear roles and responsibilities of peer support workers during the development of peer support services. This process necessitates training both professionals and peer support workers to ensure consistent understanding within teams. Such efforts will facilitate effective collaboration between peer support workers and other professionals. Additionally, providing supervisory and support networks is crucial for fostering the development of peer support services [50, 53–55]. Actions are needed to persuade the government to allocate funds and legislate to promote peer support services within Taiwan’s mental health sector.

While the first three subthemes related to environmental factors were more frequently discussed in both Western and non-Western literature [24, 50, 52], the fourth subtheme – the only one related to personal factors – was less frequently addressed. Our participants emphasized the importance of fostering self-awareness and a sense of responsibility among people with mental illness in Taiwan. These individuals, who often receive psychiatric rehabilitation services in a paternalistic and protective manner, may struggle to express their thoughts and make decisions. Unlike peer support services in Western countries, which are typically initiated by people with mental illness themselves, those in Taiwan have been introduced through the efforts of professionals, scholars, and grassroots organizations. These stakeholders have drawn insights from Western literature and international site visits. Taiwanese people with mental illness often lack familiarity with the concept of recovery and empowerment, and possess a limited understanding of peer support services. While some self-organized groups have emerged among this population in recent years, individuals serving as role models or advocates remain relatively rare. Offering courses to enhance the understanding of recovery and self-awareness among people with mental illness could be an initial step in expanding the pool of potential peer support workers. Two structured recovery programs are available in Taiwan: the Illness Management and Recovery program [56, 57] and the Grow to Recovery program [58]. These programs can provide a foundation for peer support worker training. It is worth noting that compared to Western contexts, people with mental illness in Taiwan may require more time to grasp and internalize these concepts [58]. However, with time, they may gain increased confidence in assuming the role of peer support workers.

The study encountered several limitations. First, participants were drawn from well-established and recovery-oriented psychiatric rehabilitation organizations in Taiwan, potentially limiting the transferability of the findings to professionals associated with traditional psychiatric rehabilitation organizations, clubhouses/quasi-clubhouses or diverse cultural contexts. In addition, since peer support services are still in their early stages in Taiwan, and only one collaborating organization has implemented a peer support training program, most participants had limited experience with these services. This limited exposure may have constrained their perspectives on peer support. Moreover, the perspectives of other key stakeholders, such as people with mental illness and their family members, were not included in the study, resulting in a more limited scope of the findings. Nevertheless, there is a dearth of literature examining the viewpoints of professionals in non-Western contexts regarding the concept of recovery and peer support. To address this gap, the study intentionally recruited professionals from various backgrounds, locations, and settings within Taiwan, aiming to provide a comprehensive perspective and contribute preliminary insights to this emerging field.

## Conclusions

The study revealed that professionals from well-established psychiatric rehabilitation organizations in Taiwan generally acknowledged most recovery principles, which indicated the feasibility of recovery in non-Western contexts. However, inconsistencies were found regarding the applicability of recovery, even among professionals familiar with the concept. It is recommended that professionals in non-Western contexts undergo additional practical training to effectively provide services for individuals with diverse conditions. In addition, peer support was often used informally in psychiatric rehabilitation organizations in Taiwan. Participants identified both environmental and personal factors that need to be considered in developing peer support services. In particular, people with mental illness in non-Western contexts may encounter more challenges in becoming peer support workers, requiring additional training to foster self-awareness and empowerment. There is an urgent need to advocate for government support to establish relevant policies and training programs, and to integrate peer support workers as key partners in the recovery journey for people with mental illness in Taiwan.

## Data Availability

The datasets generated and/or analyzed during the current study are not publicly available due to the protection of participants’ privacy but are available from the corresponding author on reasonable request.
